# Supplementary figures and images for: Lipidomic QTL in Diversity Outbred mice identifies a novel function for α/β hydrolase domain 2 (Abhd2) as an enzyme that metabolizes phosphatidylcholine and cardiolipin
Source: PLoS Genet. 2023 Jul 31;19(7):e1010713. doi: 10.1371/journal.pgen.1010713 (PMC10414554; doi:10.1371/journal.pgen.1010713)

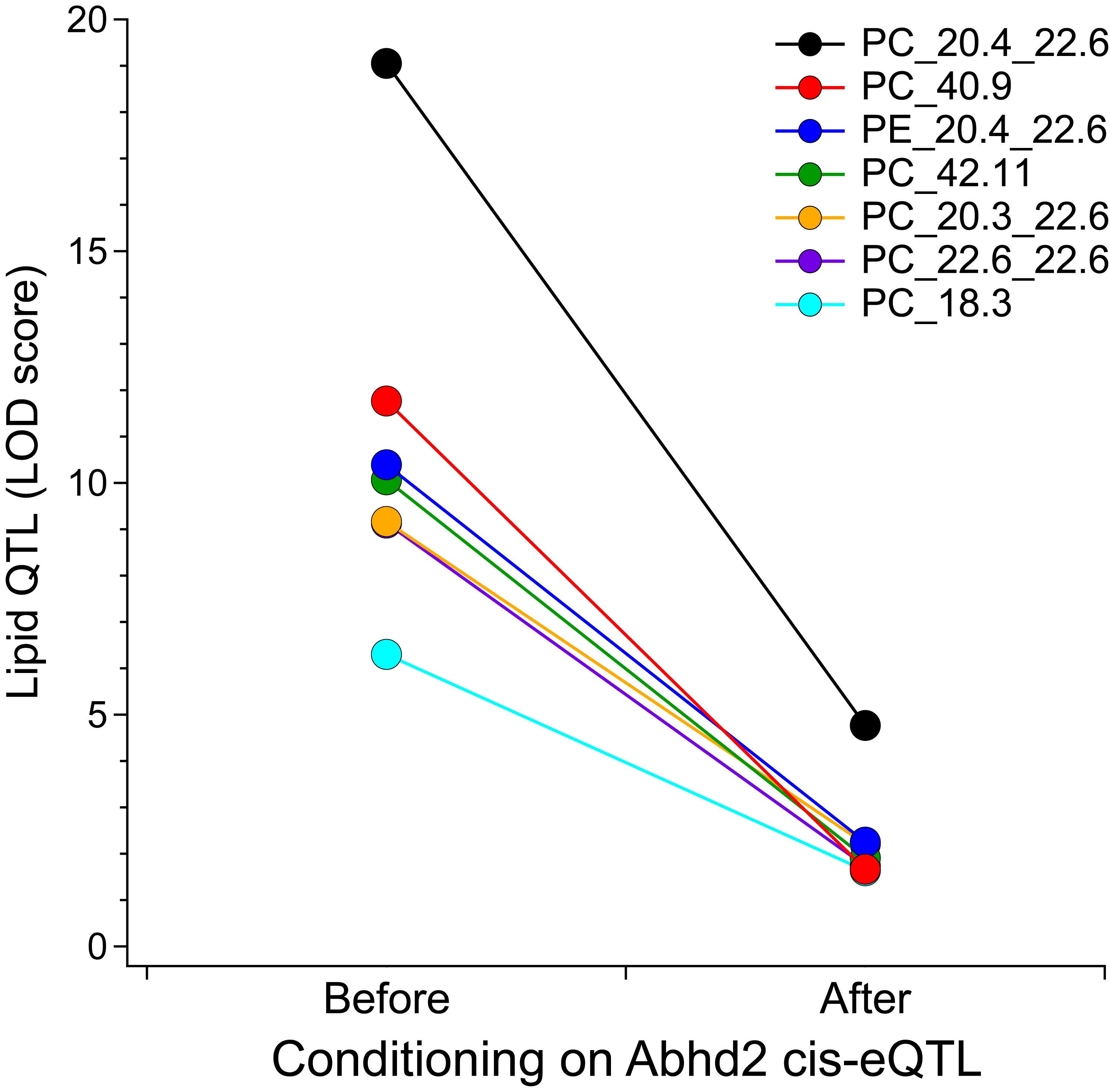

Supplement: S1 Fig — Mediation analysis of the QTL for seven liver lipids that map to the chromosome 7 locus resulted in substantial LOD drop when conditioned on hepatic Abhd2 expression. Conditioning on all other genes did not result in any appreciable LOD drop for these lipids. (TIF) [file pgen.1010713.s001.tif]

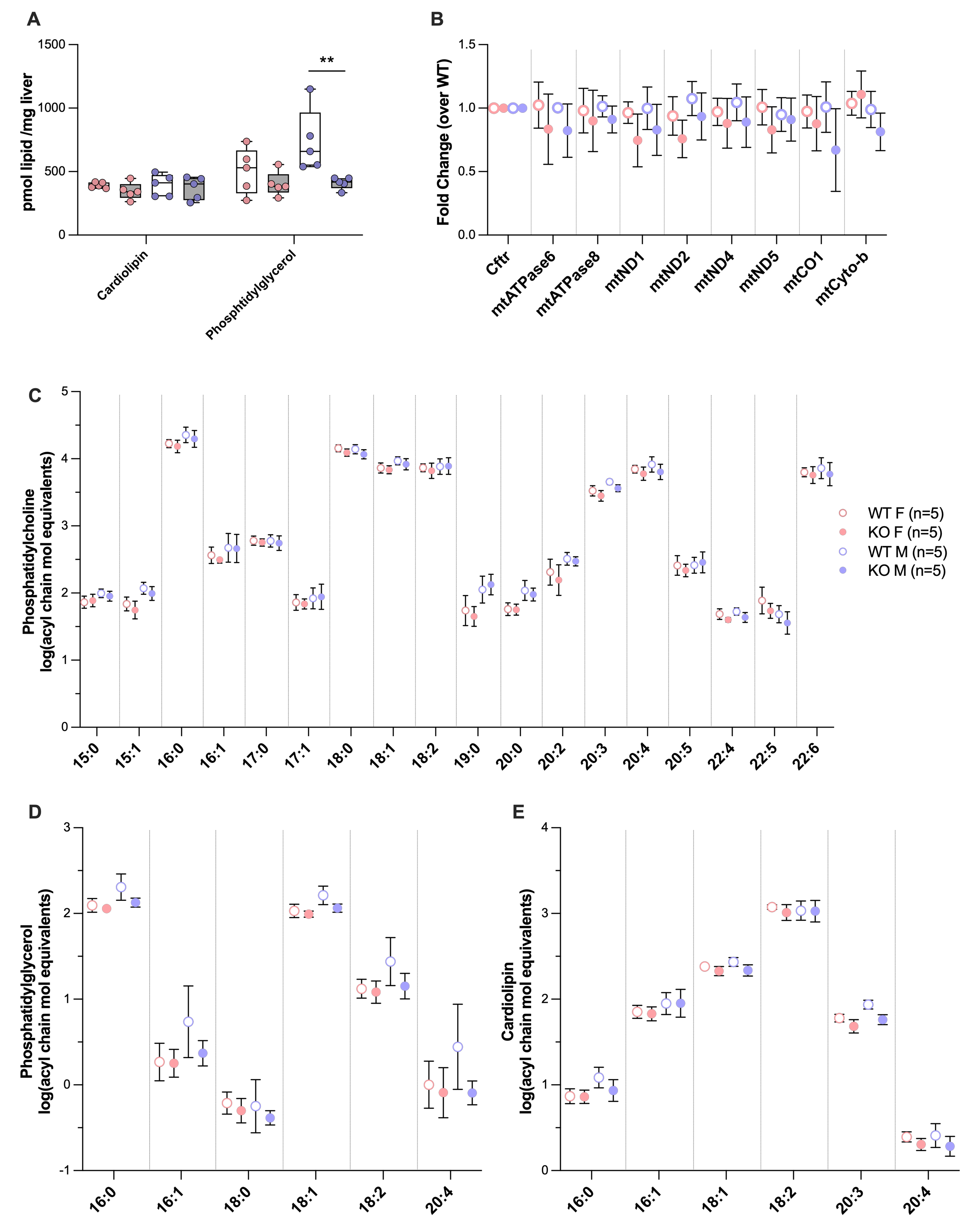

Supplement: S2 Fig — (A) Despite significant reductions in several cardiolipin species in male mice, total hepatic cardiolipin levels in male and female mice did not differ by genotype. However, total phosphatidylglycerol concentrations were decreased Abhd2KO mice compared to WT males (p<0.01). (B) Mitochondrial gene expression, measured as a proxy for mitochondrial number, was not different by sex or genotype. Neither genotype nor sex affected fatty acyl composition of PC, PG or CL in the livers of HF/HS-fed mice. (C) The hepatic phosphatidylcholine landscape was diverse and primarily comprised of acyl chains of C16 or C18 in length and were saturated or monounsaturated. (D) Phosphatidylglycerols were equally represented by fatty acyl lengths of C16 and C18 and contained 0 or 1 double bond. (E) Cardiolipins were highly represented by linoleate, with C18 being 95% of acyl lengths and 98% of CLs containing 1 or more double bonds. (TIFF) [file pgen.1010713.s002.tiff]

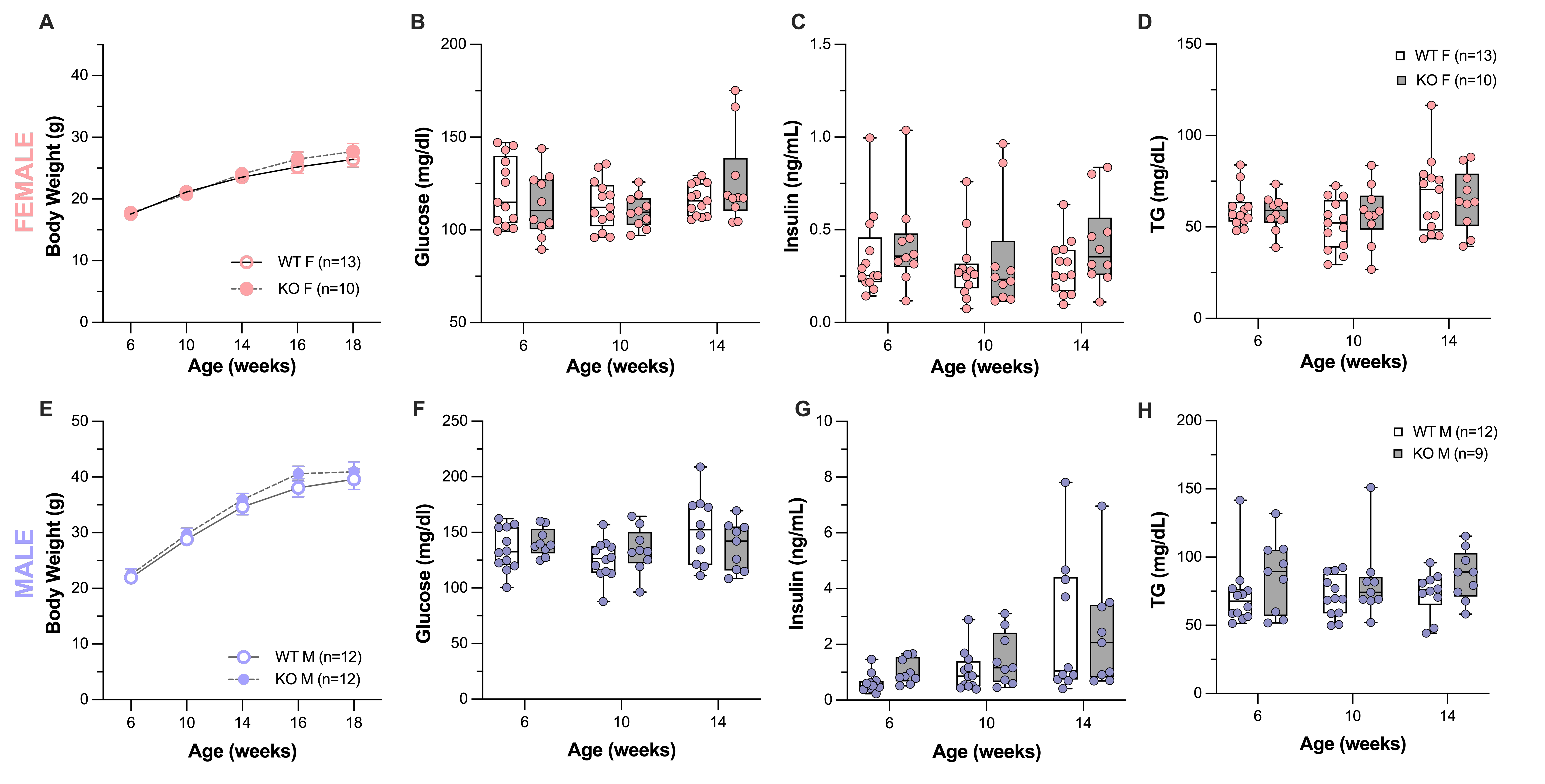

Supplement: S3 Fig — Abhd2KO female (A) and male (E) mice showed similar growth curves to WT mice. Fasting glucose (B, F), insulin (C, G), and triglycerides (D, H) did not differ by genotype. (TIFF) [file pgen.1010713.s003.tiff]

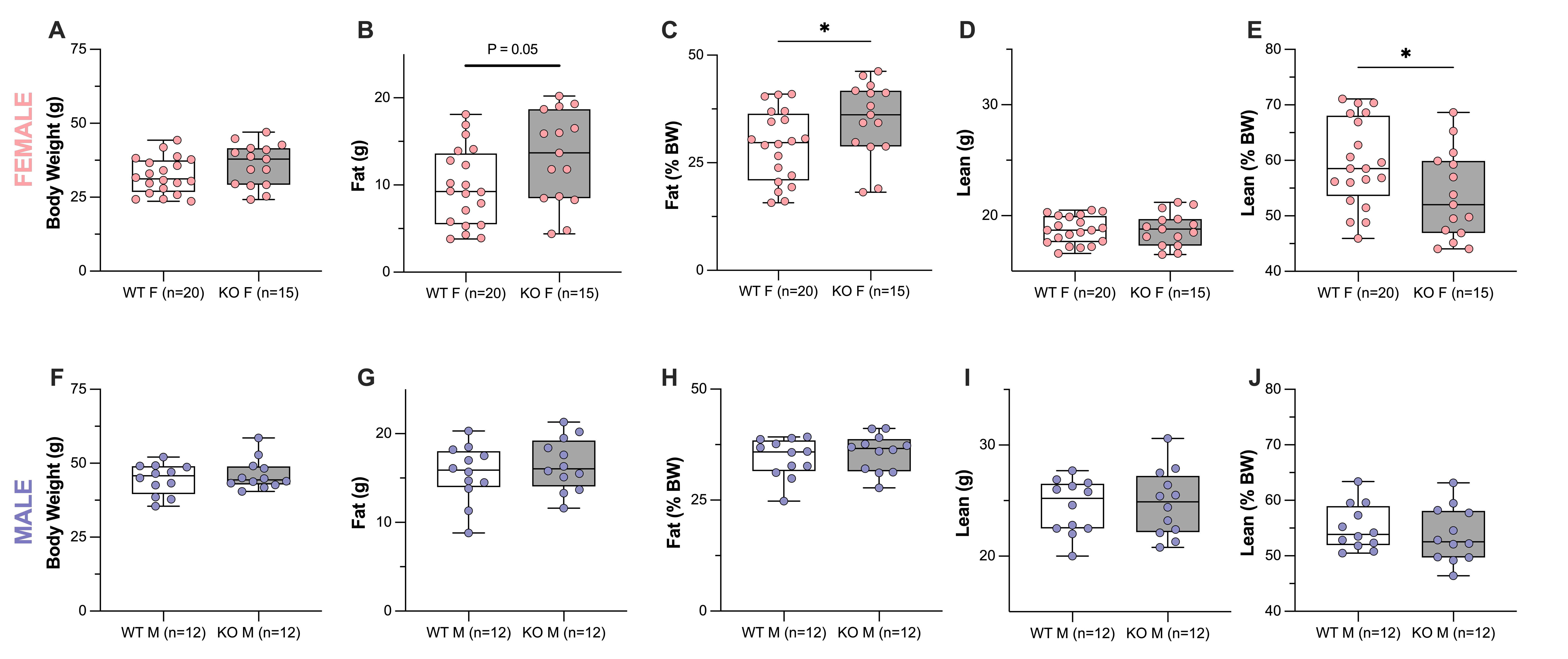

Supplement: S4 Fig — Body compositions of mice were measured at ~24 weeks of age by DEXA. (A) Body mass of female mice were not significantly different. Fat mass, both as total weight (B) and %body weight (C) increased in Abhd2KO female mice. Lean mass weight (D) did not change with genotype in females, but lean mass as %body weight (E) was reduced in Abhd2KO female mice. Male mice were not different in total body weight, fat, nor lean mass (F-J). *p<0.05. (TIFF) [file pgen.1010713.s004.tiff]

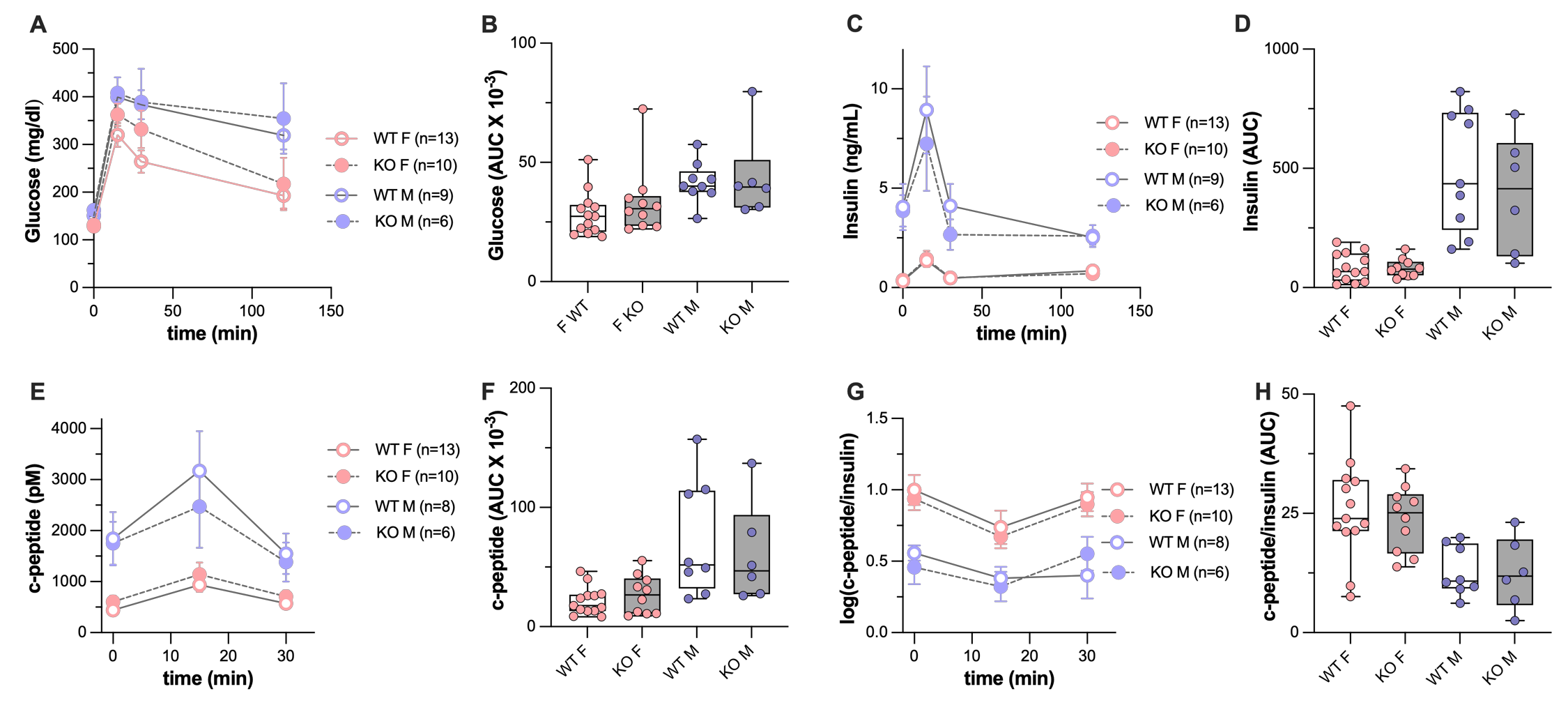

Supplement: S5 Fig — (A) Female Abhd2KO mice showed a trend for increased plasma glucose at 15 and 30-minute timepoints during the oGTT. Male Abhd2KO mice were not different. (B) Area under the curve (AUC) for plasma glucose during the oGTT did not differ by genotype. (C) Plasma insulin response to glucose stimulation were the same for genotypes of each sex, with all mice returning to baseline within two hours of receiving the glucose bolus. (D) Insulin curve AUCs were not different. (E) C-peptide, a marker of insulin secretion, was the same for genotypes of each sex during the oGTT, with no difference in AUC (F). (G) The C-peptide/insulin ratio, used as a surrogate for insulin clearance, were not different at 0, 15, and 30 minutes. (H) AUCs for C-peptide/insulin ratio were similar between genotypes of the same sex. (TIFF) [file pgen.1010713.s005.tiff]

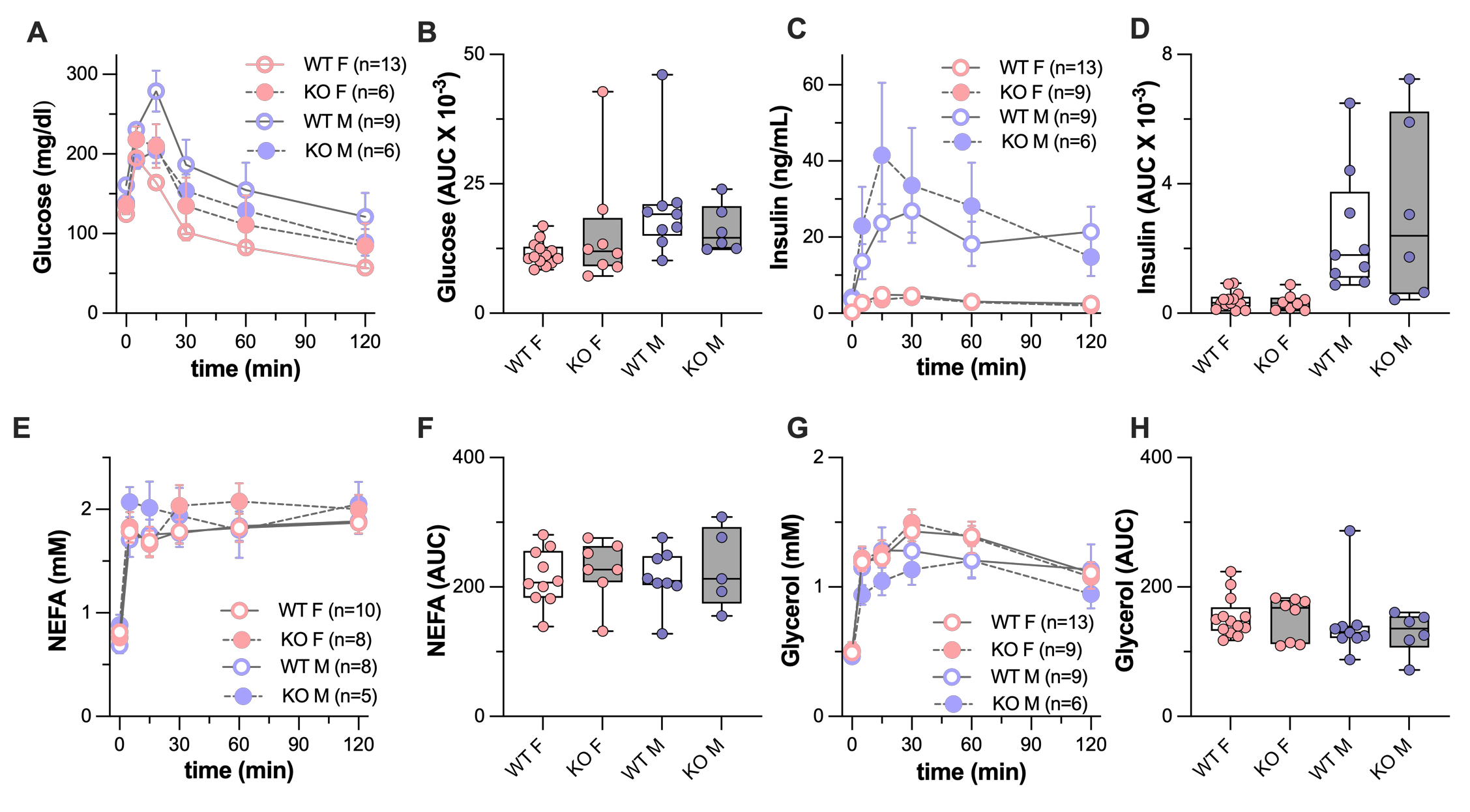

Supplement: S6 Fig — Plasma glucose concentrations at various time points (A) and total AUC for glucose (B) during β3-adregeneric receptor agonist stimulation was not different in male or female Abhd2KO mice. Plasma insulin concentrations (C) and total AUC for insulin (D) during the B3TT were the same for genotypes of each sex. Non-esterified fatty acid (NEFA) concentration (E) and total AUC for NEFA (F), and glycerol concentration (G), and AUC for glycerol (H) during the β3TT did not different for Abhd2KO female or male mice. (TIFF) [file pgen.1010713.s006.tiff]

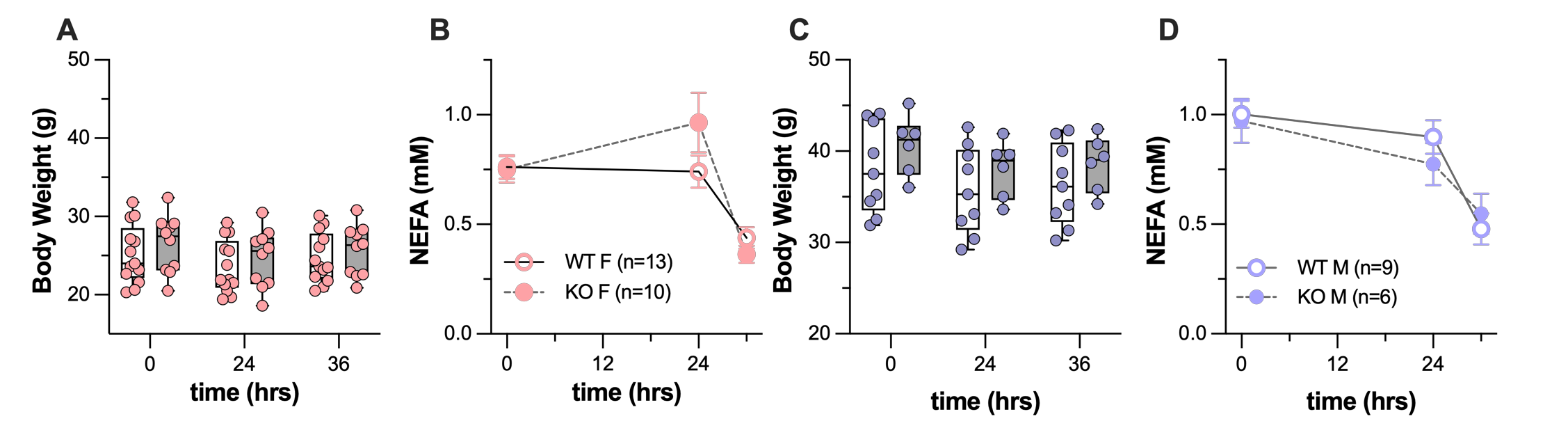

Supplement: S7 Fig — Following a 24-hr fast, female mice averaged a 1.7 ± 0.9 gm weight loss and an average 1.1 ± 0.1 gm weight gain following the 6-hour refeed period and were not different for Abhd2KO versus WT mice (A). Plasma NEFAs, measured before and after prolonged fast, were similar between genotypes (B). Male mice lost 2.5 ± 0.2 gm with prolonged fasting and regained 0.6 ± 0.1 gm following refeeding and were not different between genotypes (C). Plasma NEFAs of male mice during the fast/refeed protocol did not differ by genotype (D). (TIFF) [file pgen.1010713.s007.tiff]

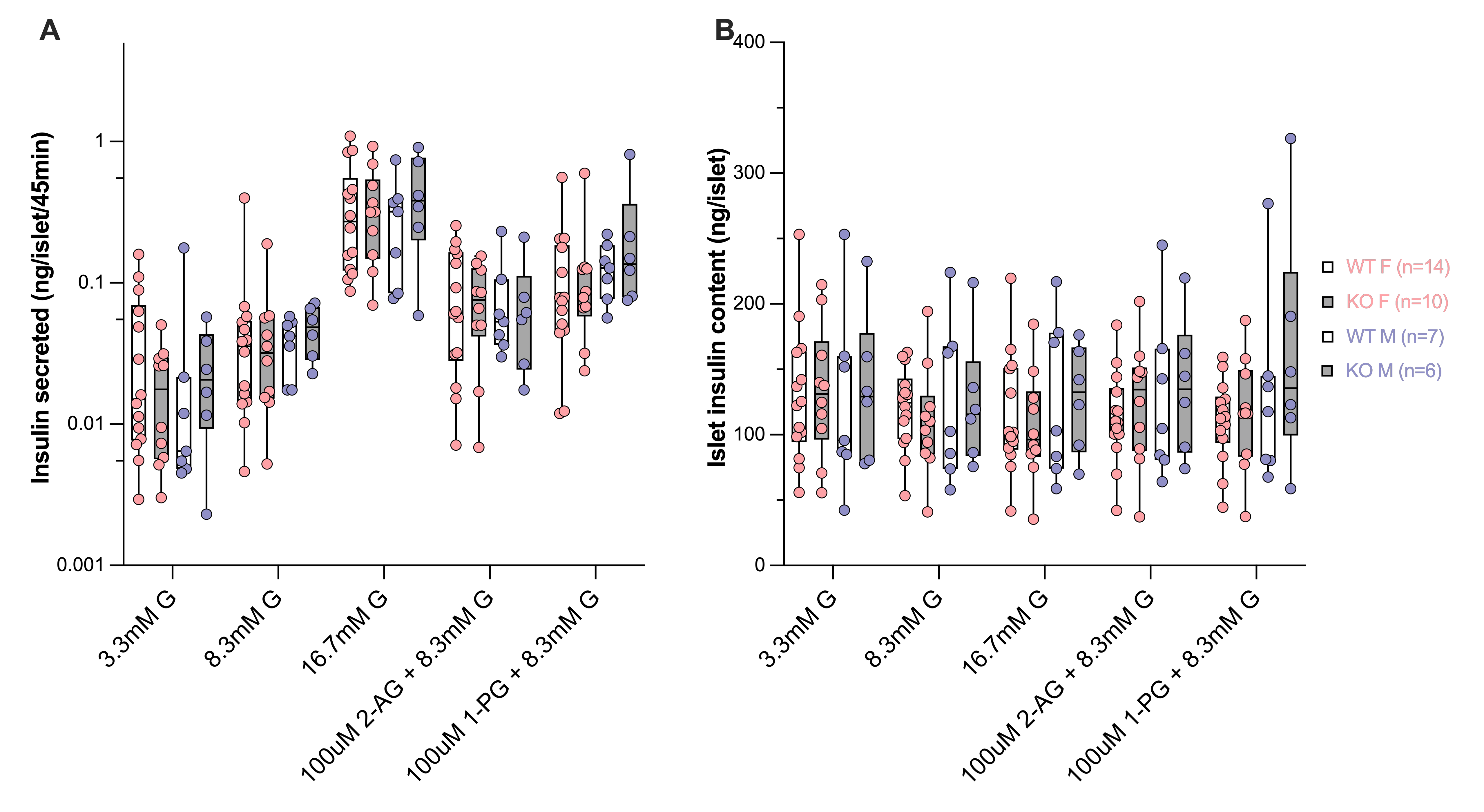

Supplement: S8 Fig — Insulin secretion in response to varying glucose concentration, or two different monoacyl-glycerols (2-AG or 1-PG) (A) and total islet insulin content (B) remained unchanged in cultured islets from female and male Abhd2KO versus WT mice. (TIFF) [file pgen.1010713.s008.tiff]

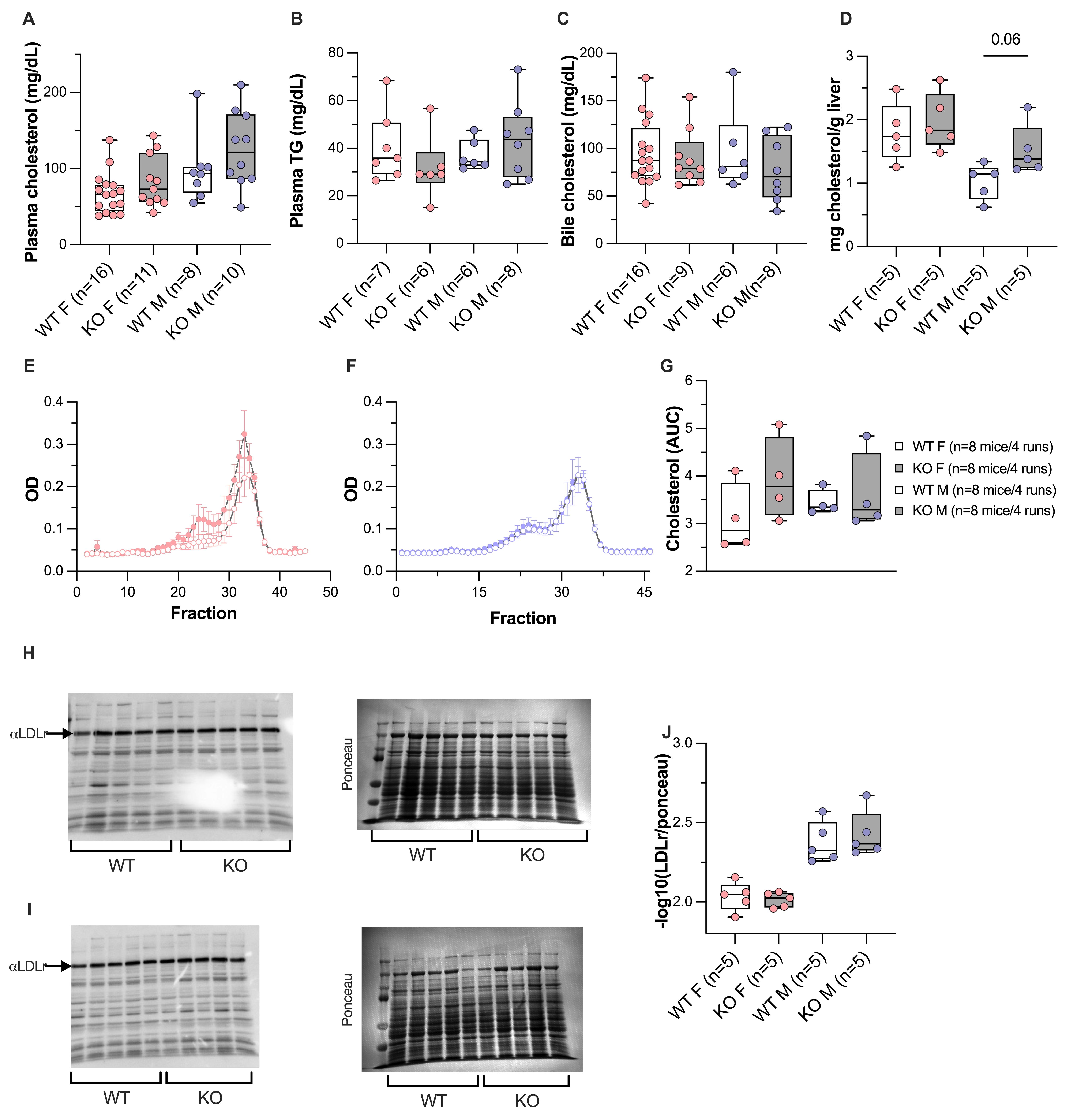

Supplement: S9 Fig — Total plasma cholesterol (A) and triglycerides (B), biliary cholesterol (C), and hepatic cholesterol (D) in female and male Abhd2KO versus WT mice. Male Abhd2KO mice showed a small increase in hepatic cholesterol (p = 0.06). Plasma cholesterol lipoproteins were separated by FPLC and assayed for cholesterol in female (E) and male (F) mice. Total AUC for cholesterol in all FPLC fractions (G). Liver from female (H) and male (I) mice were analyzed for LDL-receptor (LDLR) protein content by immunoblot. (J) Quantitation of LDLR protein abundance was not different between genotypes of the same sex. (TIFF) [file pgen.1010713.s009.tiff]
