# Supplementary material for: Lipidomic QTL in Diversity Outbred mice identifies a novel function for α/β hydrolase domain 2 (Abhd2) as an enzyme that metabolizes phosphatidylcholine and cardiolipin
Source: PLoS Genet. 2023 Jul 31;19(7):e1010713. doi: 10.1371/journal.pgen.1010713 (PMC10414554; doi:10.1371/journal.pgen.1010713)
Supplement: Attachment — Submitted filename: Reviewers_Plos Genet.docx [file pgen.1010713.s019.docx]

This study by Price et al identifies Abhd2 as a susceptibility gene for phospholipid metabolism in the liver of mice via QTL analysis, which is subsequently validated by findings in a Abhd2 mouse model. This is a relatively straightforward paper with logical progression of hypothesis and analysis. The mass spectrometry based lipidomics for a large cohort is striking and the role of Abhd2 in modulating phospholipid is well demonstrated. However, a few concerns or suggestions are noted to improve the rigor of the results and conclusions.

1) LOD threshold: Permutation tests are generally performed to determine the threshold for significant or suggestive linkage, especially when multiple covariates (sex, wave, and PC-20:4/22:6) are included. In the current study, the LOD score threshold for QTL identification was arbitrarily set at 6. Is this threshold for significant or suggestive linkage?

2) Mediation analysis to infer causality: It is stated “Mediation analysis to establish causality was performed using conditional regression of the target phenotype on gene expression of candidate gene and the locus genotype”, but results after adjustment with “the locus genotype” have not shown.

3) The genetic background of Abhd2 knockout mice was not provided.

4) Plasma cholesterol was measured, but why not phospholipids?

5) The method for measuring mitochondrial lipids should be provided.
