# Supplementary material for: Lipidomic QTL in Diversity Outbred mice identifies a novel function for α/β hydrolase domain 2 (Abhd2) as an enzyme that metabolizes phosphatidylcholine and cardiolipin
Source: PLoS Genet. 2023 Jul 31;19(7):e1010713. doi: 10.1371/journal.pgen.1010713 (PMC10414554; doi:10.1371/journal.pgen.1010713)
Supplement: Attachment — Submitted filename: Abhd2_ResponsetoReviewers.docx [file pgen.1010713.s020.docx]

Dear Editors,

We are pleased that the Editorial Board and Reviewers found our manuscript suitable for publication in PLOS Genetics. We appreciate the careful and thoughtful comments by the Reviewers. We have addressed all Reviewer comments and believe these revisions have strengthened our paper by refining methods and enhancing our discussion sections. Below we have detailed our responses to the individual reviewer comments. Reviewer comments are bulleted; our responses are in italics with the corresponding line number in the updated manuscript. We hope these changes result in the manuscript being acceptable to PLOS Genetics for publication.

**Reviewer 1**

- LOD threshold: Permutation tests are generally performed to determine the threshold for significant or suggestive linkage, especially when multiple covariates (sex, wave, and PC-20:4/22:6) are included. In the current study, the LOD score threshold for QTL identification was arbitrarily set at 6. Is this threshold for significant or suggestive linkage?
  - *Permutation testing was performed and established a suggestive threshold at LOD = 6.0 and significant threshold at LOD = 7.5. We have updated the methods section to include this information. We have also added citations for the methods used for threshold cutoffs. Lines 437-440*
- Mediation analysis to infer causality: It is stated “Mediation analysis to establish causality was performed using conditional regression of the target phenotype on gene expression of candidate gene and the locus genotype”, but results after adjustment with “the locus genotype” have not shown.
  - *Mediation analysis is performed by regression analysis of the phenotype on the locus genotype to establish a direct genetic effect. Then, candidate genes were included as covariates in the regression analysis. If the phenotype-genotype association was no longer significant, the gene was considered to be a mediator. We recognize that the original methods statement was confusing and have now amended the text to be more clear. Lines 441-445*
- The genetic background of Abhd2 knockout mice was not provided.
  - *This was an oversight on our part and the corresponding text has now been amended. Abhd2 mice are on a C57Bl/6J background. Line 455-459*
- Plasma cholesterol was measured, but why not phospholipids?
  - *We thank the reviewer for this comment and acknowledge that the lack of plasma phospholipid measurements can be a limitation to our study. In brief, we obtained a very small volume of plasma from our mice and had to prioritize its utilization both in absolute quantity and cost of reagents. Plasma from mice is a finite quantity and we chose to prioritize plasma cholesterol measures over plasma phospholipids. Our rationale for this was that if changes in liver phospholipids were significantly changing in a manner that would affect plasma cholesterol/lipoprotein metabolism, it was more important to obtain a direct measure of this outcome rather than total plasma phospholipids.*
- The method for measuring mitochondrial lipids should be provided.
  - *Mitochondrial lipids were included in the untargeted mass-spec lipidomics survey and were not measured separately. Methods section: liver lipidomics (Lines 485-505)*

**Reviewer 2**

- The study could benefit from a more detailed discussion of the limitations, such as the lack of mechanistic insight into how Abhd2 regulates phospholipid levels and the need for further investigation into tissue-specific roles of Abhd2 in lipid metabolism.
  - *We thank the reviewer for this comment and have added limitations to our discussion section. Lines 400-414*
- Given that Abhd2 has been identified as a candidate gene in human GWAS studies (https://www.ebi.ac.uk/gwas/genes/ABHD2) for lipid-related diseases, such as age-related macular degeneration (as reported in the paper by Persad et al., 2017), it would be valuable for the authors to discuss the potential relevance of their findings to these diseases.
  - *Thank you for bringing this study to our attention. We had previously looked at human GWAS for metabolic traits and only found an association for CAD near the Abhd2 locus which others showed to be attributed to a different gene. Having now considered study mentioned by Reviewer 2, we have included a small discussion of Abhd2's potential role in age-related macular degeneration. We did not collect eye tissue nor measure any macular degeneration phenotypes. However, we have added 4 sentences where we speculate that Abhd2 deficiency, through its effects on CL, may contribute to AMD. Lines 373-380*
- Consider mentioning the mouse reference genome build used in the study to ensure reproducibility and clarity for future research.
  - *Genome build is GRCm38 and gene annotation is Ensembl Ver 75. The text has now been amended to include this information. Lines 432-433*
- I suggest including more details about how the threshold for significance was determined for R/QTL2 analysis. This could include information about the rationale behind the choice of a LOD score greater than 6.0 as the threshold for identifying a QTL.
  - *Thank you for this comment. We have now expanded our methods to include rationale for LOD thresholds and citations to our prior work that established suggestive significance at LOD = 6.0 and significant threshold at LOD = 7.5. Lines 437-440*

**Reviewer 3**

- Figure 1B: It will facilitate the understanding of the readers if the authors write the genotypes of the alleles versus Abhd2 mRNA and phospholipid levels.
  - *The genotypes of the alleles (associated strains) are on the top x-axis of Figure 1B's heatmap. Figure 1B caption (line 565-566)*
- Line 124, can the authors include a sentence about the significance of the observation. Something like “genetic architecture, suggesting that Abhd2 could participate in PCs degradation”.
  - *We have added a sentence to* *highlight that the inverse genetic architecture is indicative of a “substrate” signature, suggesting that Abhd2 participates in the phospholipid’s degradation. Lines* 127-129
- Line 132: Please explain what mediation analysis is. This will facilitate the understanding of the readers.
  - *A simple explanation of mediation analysis is included in the sentences following the one originally on line 132. We have reworked our paragraph to improve clarity and have also added a more detailed explanation to our methods section. Lines 139-143; 441-445*
- Figure 2 could be supplementary. It supports the findings observed in Fig 1.
  - *We agree with this comment. Figure 2 has been moved to Supplementary Figure S1. We have also updated figure numbers in main text and figure captions to reflect the new figure numbers*
- Figure 3: Lipidomics in Abhd2 ko: In what genetic background is the Abdh2 ko, and how is Abhd2 expressed in this strain? This aspect is relevant because the consequences of its deletion could be different if it is in a highly expressed tissue or a low expression tissue.
  - *This was an oversight on our part and the corresponding text has now been amended to include the genetic background. Abhd2 mice are on a C57BL/6J background. Line 459*
  - *Our lab has published liver transcriptomics of the 8 founder strains (data available at http://diabetes.wisc.edu/cc_founder.php). Abhd2 is highly expressed in liver tissue of all the founder strains on a HF/HS diet. With this expression level in liver, we were confident in using the Lishko lab's Abhd2 KO mice on a C57BL/6 background in testing the physiological role of Abhd2. Abhd2 is also highly expressed in adipose and islet, which is why we sought to investigate potential roles in insulin secretion, adiposity, and lipolysis.*
- In what subcellular compartments are these lipids stored? Do the Abhd2 ko show features of a Lysosomal Storage Disorder (LSD)? Could Abhd2 be a modifier gene of phospholipidosis? Can the authors show electron microscopy images of the tissues or discuss this point?
  - *Phosphatidylcholine is relatively evenly distributed throughout the cell, comprising ~50% of lipids in the plasma membrane and most organelles (PMID: 29588369). Phosphatidylethanolamine is the second most abundant phospholipid in the plasma membrane and organelles and is similarly distributed throughout the cell (~20% of organelle phospholipids). CL is exclusively in the mitochondria and mostly in the inner mitochondrial membrane.*
  - *The suggestion of lysosomal storage disorder is interesting and one that we had not considered. While we did not observe characteristic muscle wasting or sphingomyelin/cholesterol accumulation in liver tissue, we did not perform electron microscopy to evaluate lysosomes directly. We have added this as a limitation of our study. Lines 405-411*
- Gene expression of mitochondrial genes does not say anything about its functionality. Can the authors show any functional data of the mitochondria?
  - W*e thank the reviewer for this insightful comment and agree that mitochondrial DNA does not speak to functional measures. We assessed mitochondrial DNA as a marker of mitochondrial number in Abhd2KO livers. We did not observe a change in mtDNA and thus believe that mitochondrial number is not changed after deletion of Abhd2. However, we agree mitochondrial function should be assessed in future studies of Abhd2.*
- Can the authors expand the discussion about the sex-specific lipid changes in the Abdh2 ko? Does the Abdh2 promotor has an estrogen-responding element?
  - *Abhd2 does not have a ERE binding sequence (as defined by PMID: 9792632); previous studies by the Lishko lab have established Abhd2 as having a progesterone binding site which is required for its function in reproductive physiology (PMID: 26989199).*
- What is the sex of the animals where the initial screening was performed?
  - *The initial DO screen included both sexes at ~50% M/F. Of the total 384 animals used in liver lipid QTL analysis, 191 were female and 193 were male. We have added this information to the methods section. Line 429*
- Figure 1B: it is difficult to interpret the results how they are currently plotted. Can the authors show the correlations between the gene expression of Abh2 and Abh18 genes across the different DO parental strains? Do Abh2 and Abh18 co-localized? Please show a confocal image to support that they could be working together. Is any data predicting their physical interactions?
  - *Due to poor antibody quality, we cannot show confocal images. Through subcellular fractionation and western blotting, others have shown Abhd2 localized to the ER (PMID: 28684316). As mentioned in our discussion, Abhd18 was found in mitochondrial proteomics, but Abhd2 was not annotated in the same study (PMID: 34800366). The String protein interaction database predicts an interaction between Abhd2 and Abhd18. However, we cannot provide empirical data/images to prove (or disprove) this predicted interaction.*
- In vivo physiological measurements: Did the authors measure liver function tests, such as alanine transaminase (ALT) and aspartate transaminase (AST), alkaline phosphatase (ALP), gamma-glutamyl transferase (GGT), among others?
  - *We did not measure any liver functional enzymes or assess liver function. In this study, we focused on liver lipids and common metabolic pathways associated with altered phospholipid biology.*
